# Intermittent hyperglycaemia induces macrophage dysfunction by extracellular regulated protein kinase‐dependent PKM2 translocation in periodontitis

**DOI:** 10.1111/cpr.13651

**Published:** 2024-05-24

**Authors:** Yuezhang Sun, Aimin Cui, Hao Dong, Lulingxiao Nie, Ziqi Yue, Jiao Chen, Wai Keung Leung, Jian Wang, Qi Wang

**Affiliations:** ^1^ State Key Laboratory of Orval Diseases, National Center for Stomatology, National Clinical Research Center for Oral Diseases, Department of Prosthodontics, West China Hospital of Stomatology Sichuan University Chengdu China; ^2^ Periodontology and Implant Dentistry Division, Faculty of Dentistry The University of Hong Kong Hong Kong China; ^3^ Sichuan Provincial Engineering Research Center of Oral Biomaterials, Sichuan University Chengdu China

## Abstract

Early fluctuations in blood glucose levels increased susceptibility to macrophage dysfunction. However, the underlying pathological mechanisms linking glucose variations and macrophage dysregulation remains elusive. In current study, we established an animal model of transient intermittent hyperglycaemia (TIH) to simulate early fluctuations in blood glucose levels. Our findings revealed that both TIH and diabetic group exhibited more severe periodontal lesions and increased secretion of pro‐inflammatory cytokines compared to healthy controls. In immortalized bone marrow–derived macrophages (iBMDMs), phagocytosis and chemotaxis were impaired with transient and lasting hyperglycaemia, accompanied by enhanced glycolysis. We also found that TIH activated pyruvate kinase M2 (PKM2) through the phosphorylation of extracellular regulated protein kinase (ERK) in vivo, particularly at dimeric levels. In macrophage cultured with TIH, PKM2 translocated into the nucleus and involved in the regulating inflammatory genes, including TNF‐α, IL‐6 and IL‐1β. PKM2 translocation and secretion of inflammatory cytokines were attenuated by PD98059, while PKM2 tetramer activator TEPP‐46 prevented the formation of dimeric PKM2 in macrophages. Moreover, inhibition of glycolysis alleviated the TIH‐induced pro‐inflammatory cytokines. In conclusion, our manuscript provides a rationale for understanding how TIH modulates metabolic rewiring and dysfunction in macrophages via ERK‐dependent PKM2 nuclear translocation.

## Graphical abstract

Glucose variation initiated ERK‐dependent PKM2 nuclear translocation, leading to enhanced aerobic glycolysis and macrophages dysregulation. Our findings indicate that PKM2 represents a potential therapeutic target for immune‐metabolism in inflammation.

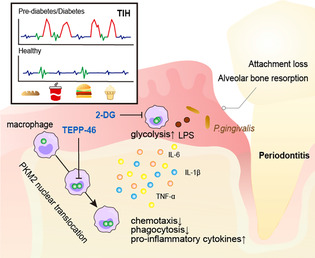

## 1 INTRODUCTION

Macrophages play a pivotal role in the pathophysiological mechanisms of metabolic disorders and its associated complications.^1^, ^2^ Hyperglycaemia disrupts macrophage functions through inflammasome‐activation and secretion of senescence‐associated secretory phenotype.^3^, ^4^, ^5^ The accumulation of metabolic changes and impaired macrophages function perpetuates persistent inflammation, even when conventional therapies are employed to maintain blood glucose levels within the normal range. The phenomenon of hyperglycaemic “legacy effect” has been observed in clinical and basic researches.^6^, ^7^ Most patients would experience abnormal fluctuations of glycaemia before diagnosed as diabetes, and blood glucose fluctuations may continue in some of them after receiving treatment.^8^ Several studies have indicated that macrophages can retain previous phenotype and function through epigenetic modification and metabolic reprogramming.^9^ It is worth noted that the activation of macrophages differs from adaptive memory as it is not antigen‐specific, but rather based on the reinforcement of the innate response upon subsequent encounters with pathogens^10^

Pyruvate kinase (PK) is a critical limiting glycolytic enzyme that influences metabolic activity. The distribution of the four isoforms of PK varies across specific cell types. While PKM1 is consistently expressed in most tissues, M2 is predominantly found in proliferating and tumour cells.^11^ Distinct from other isoforms, PKM2 exists in tetrameric or dimeric configurations with different biological activities. Several studies have demonstrated that dimeric PKM2 exhibits reduced metabolic activity, however, it can translocate into the nucleus and serve as a nuclear transcriptional co‐activator, thus to regulate inflammatory cytokines production in macrophages.^12^ Previous research revealed that immune cells would undergo metabolic reprogramming to fulfil their energy and biosynthetic requirements, particularly during cell activation and proliferation.^13^ PKM2 is a key factor in the modulation of cellular metabolism in mammals and plays a pivotal role in the regulation of immune cell function. PKM2‐dependent metabolic reprogramming converts towards aerobic glycolysis, commonly known as the Warburg effect observed in tumour cells. Inhibition of PKM2 translocation significantly impacts the synthesis of inflammatory factors, such as tumour necrosis factor‐alpha (TNF‐α) and interleukin‐1β (IL‐1β)^14^ It has been demonstrated that post‐translational modifications including phosphorylation, acetylation and oxidation regulate the function of PKM2.^15^, ^16^ Specially, ERK‐mediated S37 phosphorylation activates dimeric PKM2 translocation into the nucleus.^17^

In our study, we emphasized the dysregulation of macrophage chemotaxis and phagocytosis induced by TIH, which leads to an exaggerated destruction of periodontal tissues. The dysfunctional accumulation observed in macrophages can be attributed to alterations in metabolic patterns mediated by ERK‐dependent PKM2 nuclear translocation. Our findings indicated that targeting PKM2 could hold promise as a therapeutic approach for immune‐metabolism modulation in chronic inflammation.

## 2 MATERIALS AND METHODS

### 2.1 Animals

The 4‐week‐old male C57BL/6 mice were obtained from the Model Animal Research Center of Nanjing University (Nanjing, China), and handled in the State Key Laboratory of Oral Diseases at Sichuan University under standard housing conditions. Animal treatment was conducted in accordance with the protocol approved by the Institutional Committee for Animal Use and Care at Sichuan University (No. WCHSIRB‐D‐2020‐357). The mice were randomly divided into control mice (C group, *n* = 8), transient intermittent hyperglycaemia (TIH group, *n* = 8), and diabetic mice (D group, *n* = 8). At 6 weeks of age, the D group were injected with streptozotocin (STZ; Sigma‐Aldrich, Shanghai, China) at a dose of 55 mg/kg every day for 5 consecutive days. Continuous low dose injection of STZ is currently recognized as a highly effective and commonly utilized approach for inducing type 2 diabetes.^18^ After 10 weeks, the mice with stable hyperglycaemia (fast blood glucose >11.1 mmol/L) were assigned to D group. Control mice were treated with an equivalent volume of saline. All mice were euthanized at the end of experiments. The animal study conforms to the Animal Research: Reporting In Vivo Experiments (ARRIVE) 2.0 guidelines.

### 2.2 Transient intermittent hyperglycaemia model

The TIH model, as previously mentioned,^6^ has demonstrated its ability to simulate abnormal fluctuations in glycaemia while maintaining overall good glycaemic control (HbA1c < 7%). TIH mice were injected with glucose (2 g/kg i.p.) for 4 times at 2 h intervals, and an equal volume of 0.9% saline for control group. A glucometer (OneTouch Glucometer; LifeScan, Milpitas, CA, USA) was applied to measure glycaemia prior to and 15 min following each injection. The TIH procedure was repeated once weekly for 10 consecutive weeks. The body weight was measured every week by a scale with 0.01 g accuracy. The FBG detection was conducted in a single‐blind manner.

### 2.3 Cell lines and culture

The immortalized bone marrow‐derived macrophage (iBMDM) cell line was purchased from FuHeng Biology (FH‐032YSH). After culturing for 1 day, cells were passaged every 2 days. Cells from TG group were exposed to normoglucose (5.5 mmol/L glucose, NG) and high‐glucose (25 mmol/L glucose, HG) condition alternating every 2 h from 8:00 AM to 18:00 PM and then cultured with NG for another day. Then, the cells were stimulated with 100 ng/mL lipopolysaccharide (LPS; tlrl‐pglps, Invivogen) for 24 h. Cells were cultured in a humidified incubator containing 5% CO_2_ at 37°C.

Detailed materials and methods are provided in the Supporting information.

### 2.4 Statistical analysis

Statistical analysis was performed using GraphPad Prism 8.0 software. Data are presented as mean ± SD. The differences between groups were analysed with one‐way analysis of variance (ANOVA) followed by Tukey's multiple comparison test. A value of *p* < 0.05 was considered statistically significant.

## 3 RESULTS

### 3.1 TIH exacerbated *Porphyromonas gingivalis*‐induced periodontal inflammation in vivo

**FIGURE 1:**
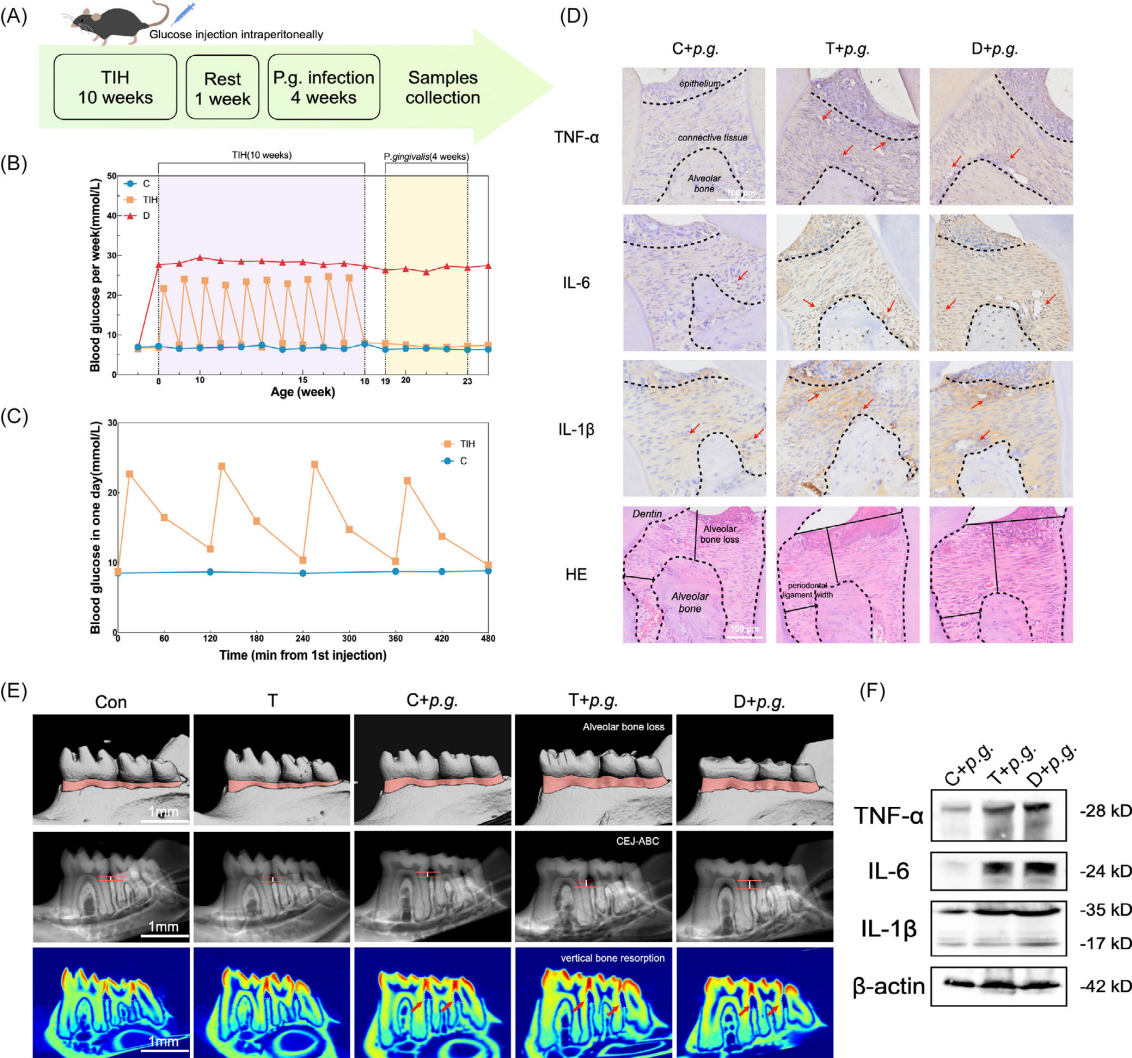
Transient intermittent hyperglycaemia (TIH) accelerates periodontal lesions in *Porphyromonas gingivalis*‐induced mice. (A) TIH protocol was performed strictly according to the procedure. (B) TIH mice were subjected to the procedure (4 injections of 2 g/kg glucose at an interval of 2 h) or saline (control) once a week for 10 consecutive weeks. C57 mice were rendered diabetic (D group) by STZ injections. (C) Fasting glucose levels were measured at 15 min and 1 h prior to and following each injection. (D) Inflammatory cytokines levels were quantified by immunohistochemistry. H&E staining shows alveolar bone loss (ABL) and periodontal ligament width in mice maxilla. Scale bar: 100 μm. (E) Micro‐CT of mandibular bones show ABL, attachment loss and vertical bone resorption. Scale bar: 1 mm. (F) Western blot demonstrated TNF‐α, IL‐6 and IL‐1β levels in mice gingiva tissues. Con, normoglycaemic mice; T, transient intermittent hyperglycaemic mice; C + *P.g*., *P. gingivalis* infection‐normoglycaemic mice; T + *P.g*., *P. gingivalis* infection – transient intermittent hyperglycaemic mice; D + *P.g*., *P. gingivalis* infection‐diabetic mice.

To explore the role of TIH in periodontal inflammatory destruction, mice were subjected to four glucose injections at 2‐h intervals once a week for consecutive 10 weeks according to the procedure (Figure 1A, B).^6^ Following each injection in TIH mice, fast blood glucose (FBG) gradually increased and peaked at approximately 15 min before slowly declining and returning to normal levels after about 2 h (Figure 1C). Before sacrifice, there was no statistically significant difference in glycaemia between TIH and control. The blood glucose of diabetic mice significantly increased and stabilized around 26 ~ 28 mM between the 8th and 24th week. Immunohistochemical (IHC) staining revealed a slight increase in the expression of pro‐inflammatory cytokines such as TNF‐α, IL‐6 and interleukin 1β (IL‐1β) within periodontal tissues of both the TIH group and D group (Appendix Figure 1A–C). Western blot analysis confirmed an enhanced expression level of these pro‐inflammatory cytokines in gingival tissue samples(Figure 1D, F; Appendix Figure 1K–M). We identified alveolar bone loss (ABL) of maxilla and mandible bone as the feature of periodontal destruction with H&E staining and micro‐CT (Figure 1D, E; Appendix Figure 1D–H). As depicted in Figure 1E, both TIH mice and diabetic mice exhibited more severe alveolar bone loss and attachment loss compared to the control after *P. gingivalis* infection. The presence of intermittent and continuous high glucose also contribute to the reduction in bone volume/total volume (BV/TV) and the increase of trabecular separation rate (Tb.Sp) in mice, indicating a decline in bone mineral density (Appendix Figure 1I,J). However, glycaemia fluctuations alone did not provoke aggressive bone loss than that in control (Appendix Figure 1F–J). The above results illustrated that TIH enhanced *P. gingivalis*‐induced periodontal inflammation in mice at both histological and molecular levels.

### 3.2 TIH up‐regulated the ERK/PKM2 expression in periodontal macrophages

**FIGURE 2:**
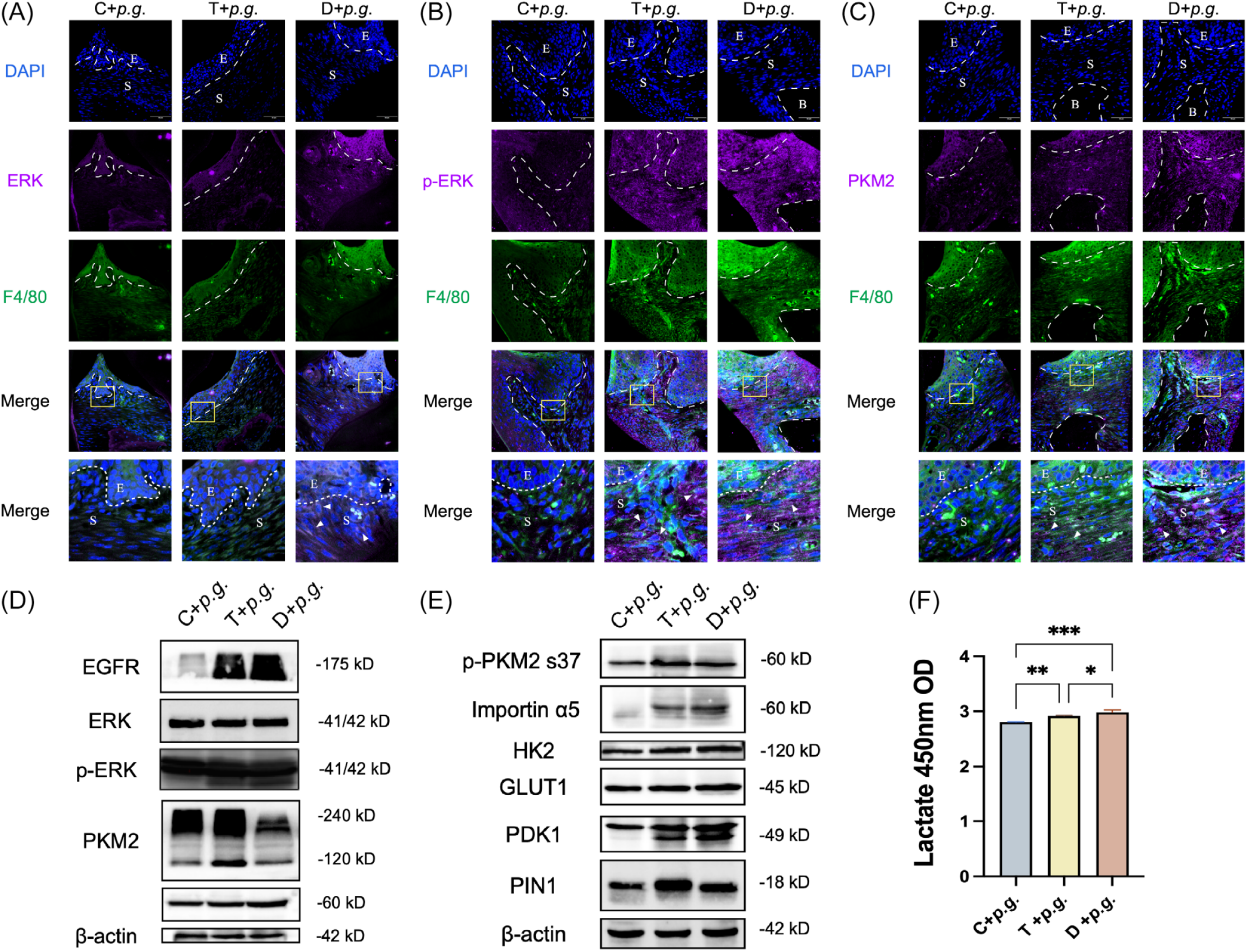
ERK/PKM2 pathway was activated in the gingival tissue of transient intermittent hyperglycaemia (TIH) mice. (A–C) ERK, p‐ERK and PKM2 levels were measured by immunofluorescence staining. IF co‐staining of ERK (purple), F4/80 (green) and DAPI (blue) in the gingival tissue of different groups. The yellow box area indicates a view of higher magnification. White arrows indicate the positive stained cells. Scale bar: 50 μm. (D) Expression levels of EGFR, ERK, p‐ERK and PKM2 in the gingival tissue of mice were detected by Western blot. (E) Expression levels of metabolic enzymes like HK2, GLUT‐1 and PDK1 in gingiva of mice. (F) Quantification of the lactate level in the serum of different groups. *p < 0.05; **p < 0.01; ***p < 0.001. Data are expressed as mean ± standard deviation (*n* = 3), repeated three times. B, alveolar bone; E, epithelium; S, subepithelial tissue. C + *P.g*., *Porphyromonas gingivalis* infection‐normoglycaemic mice; T + *P.g*., *P. gingivalis* infection‐ transient intermittent hyperglycaemic mice; D + *P.g*., *P. gingivalis* infection‐diabetic mice.

In the quest for molecular determinants responsible for TIH‐induced inflammation, we evaluated the effect of TIH on metabolism and ERK/PKM2 pathway in vivo. PKM2 serves as a critical glycolytic enzyme, as well as a nuclear transcriptional co‐activator involved in regulating inflammatory genes.^19^ Immunofluorescence (IF) assays showed the expression of p‐ERK and PKM2 increased in mice with TIH and diabetes (Appendix Figure 2A–F), while there was no significant difference observed in ERK expression among different groups (Appendix Figure 2H). Co‐staining of p‐ERK/PKM2 and F4/80 suggested enhanced expression of p‐ERK and PKM2 specifically within macrophages from TIH and diabetic mice (Figure 2A–C). Moreover, IF staining suggested more macrophages infiltration into subepithelial tissue in TIH and D group, indicating an augmented innate immune response to *P. gingivalis* infection. The negative control of IF staining of gingiva tissues and iBMDM is depicted in Appendix Figure 6. Western blot analysis yielded similar results in gingival tissue (Figure 2D; Appendix Figure 2G–L). Additionally, we observed remarkable polymerization of PKM2 into dimers and increased peptidyl‐prolyl isomerase (PIN1) in gingival tissues of T and D group, with the latter playing an essential role in facilitating PKM2 dimerization (Appendix Figure 2N,R).^11^ The results showed an increase in the phosphorylation level of PKM2 at serine 37, which is potentially targeted by ERK in mice (Appendix Figure 2M). Given the pivotal role that PKM2 plays in glycolytic metabolism, we assessed the expression levels of glycolysis‐related enzymes such as pyruvate dehydrogenase kinase 1 (PDK1), lactate dehydrogenase A (LDHA), and hexokinase II (HK2) (Figure 2E, Appendix Figure 2O–Q). HK2 catalyses the phosphorylation of glucose at the mitochondrial outer membrane, resulting in the formation of glucose‐6‐phosphate, which initiates glycolysis. The expression of HK2 and PDK1 were significantly elevated in TIH and diabetic mice, however, there was no significant distinction observed between T + *P.g*. group and D + *P.g*. group. GLUT1 can enhance glucose uptake and glycolysis rate, with slightly enhanced expression in T + *P.g*. group compared to the controlled mice. The expression level of GLUT1 increased in D + *P.g*. group compared to that observed in T + *P.g*. group and the control group. These findings suggest that persistent hyperglycaemia can induce glycolytic activation, which persists even after blood glucose levels return to normal. Our results suggest that TIH‐induced ERK/PKM2 is actively involved in the process of aggravated inflammation response, manifested by augmented anaerobic glycolysis. Subsequently, we investigated the serum lactate levels of mice, which confirmed an elevation in glycolysis activity (Figure 2F).

### 3.3 TIH restrained macrophage capability of chemotaxis and phagocytosis

**FIGURE 3:**
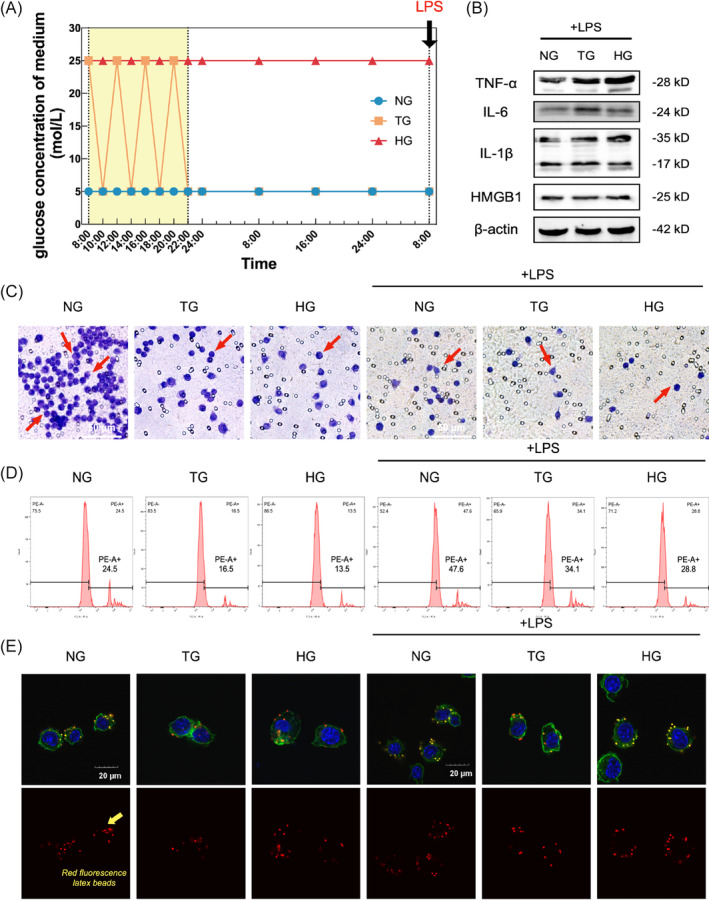
Transient intermittent hyperglycaemia (TIH) inhibits macrophage immune functions. iBMDM was treated with normoglycaemia (NG), transient hyperglycaemia (TG) and hyperglycaemia (HG). Its immune function was assessed. (A) iBMDM were cultured with normal glucose (NG), TIH (TG) and high glucose (HG) medium separately. TG medium changed every 2 h. (B) The protein expression of TNF‐α, IL‐6, IL‐1β and HMGB1 was detected by western blot. (C) Transwell migration assay revealed the chemotaxis capability. Red arrows indicate the migrated cells. Scale bar: 50 μm. (D) and (E): Flow cytometry and red fluorescence latex beads incubation demonstrated the phagocytosis function. The engulfed beads were indicated with yellow arrows. Scale bar: 20 μm. Experiments were repeated three times.

To investigate the functional and phenotypic changes in macrophages, iBMDMs were cultured with TIH or hyperglycaemia conditions (Figure 3A). The TG group was exposed to NG and HG alternating every 2 h from 8:00 AM to 18:00 PM and cultured with NG for the rest of time before LPS stimulating. We examined the expression of pro‐inflammation cytokines in vitro. As Figure 3B shows, the expression level of TNF‐α, IL‐6, IL‐1β and high mobility group box 1 (HMGB1) were elevated in TG and HG group, indicating both transient and lasting hyperglycaemia exaggerated LPS‐induced inflammation in macrophages (Figure 3B; Appendix Figure 3D–G). Furthermore, we determined the chemotaxis and phagocytosis functions with transwell migration assay and flow cytometry. The results demonstrated that TG and HG impaired both migration and phagocytosis functions (Figure 3C, D). Besides, fluorescence staining revealed a notable reduction in the capacity of macrophages to engulf latex beads with TG or HG culture medium (Figure 3E). Intriguingly, stimulation with the stimulation of LPS in all groups, while the migration cells decreased conversely (Appendix Figure 3A–C). The above results revealed the immune dysfunction of macrophage in TG and HG, thereby implying the pathogenesis of periodontal tissues impairment in TIH and diabetic mice.

### 3.4 TIH facilitates the nuclear translocation of PKM2 and regulates glycolysis metabolism in macrophages, which is indispensable for TIH‐induced macrophage inflammation

**FIGURE 4:**
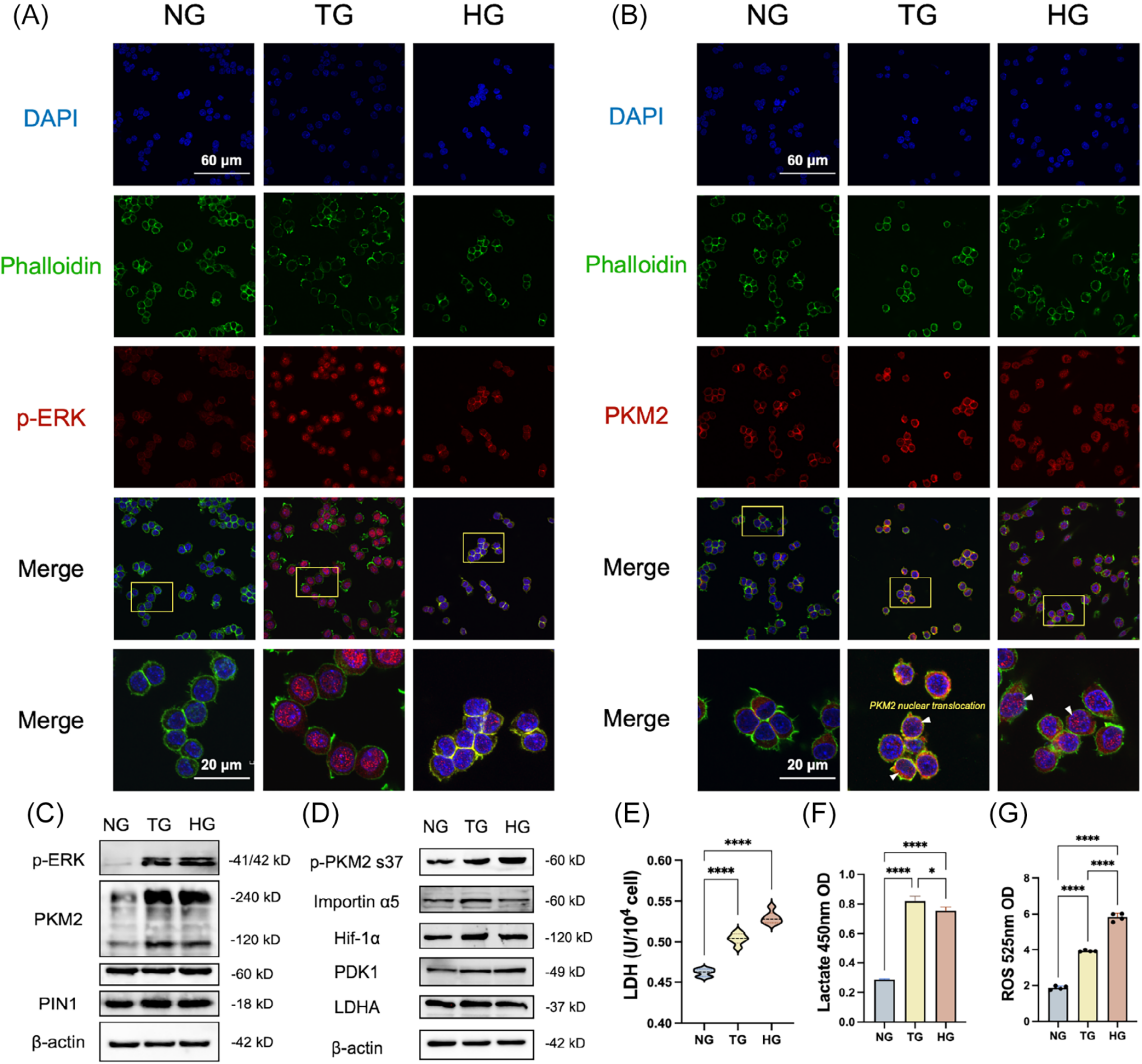
ERK/PKM2 pathway was activated in TIH‐cultured macrophages. (A, B) Immunofluorescence staining demonstrated p‐ERK and PKM2 levels. White arrows indicate the nuclear translocation of PKM2 in macrophages. Scale bar: 20 μm. (C) Western blot analysis revealed ERK, p‐ERK, PKM2 and PIN1 immunoreactivity in the iBMDM of NG, TG and HG. (D) Expression levels of p‐PKM2 s37, importin α5, Hif‐1α, LDHA and PDK1 in macrophages. (E) The lactate dehydrogenase activity was detected with an assay kit. (F) The levels of lactate were assayed using a commercial kit. (G) ROS production level was determined with a kit. Data are expressed as mean ± standard deviation (*n* = 4), repeated three times. *p < 0.05; **p < 0.01; ***p < 0.001. NG, normoglycaemia; TG, transient hyperglycaemia; HG, hyperglycaemia.

It has been revealed that the conformational and functional changes of PKM2 contribute to inflammatory advances^20^ To investigate the role of PKM2 in up‐regulated glycolysis induced by TIH, we assessed different configurations of PKM2 in macrophages in vitro. As depicted in Figure 4A, B, P‐ERK and PKM2 were activated in TG and HG condition. Moreover, the immunofluorescence analysis demonstrated a significant nuclear accumulation of PKM2, which was primarily expressed in cytoplasm in controlled macrophages, corroborating nuclear translocation of PKM2. Notably, the dimeric (120 kD) and tetrameric (250 kD) configuration of PKM2 levels were higher in macrophages exposed to TG and HG conditions (Figure 4C, Appendix Figure 4A–G). The phosphorylation of PKM2 on serine 37 was enlarged in macrophages, along with PIN1. Importin α5 functions as an adaptor for translocating PKM2 into the nucleus,^21^ and the increase of importin α5 may be associated with the accumulation of dimeric PKM2 in nucleus (Figure 4D; Appendix Figure 4H–J). Simultaneously, the expression of LDHA and PDK1 were slightly increased in TG and HG group (Appendix Figure 4L,M). The elevated lactate dehydrogenase activity and lactate production further indicated the augment of aerobic glycolysis (Figure 4E, F). Furthermore, hyperglycaemia induced an oxidative stress microenvironment within macrophages (Figure 4G). Our observation indicated that TIH or lasting hyperglycaemia affects the manner of glucose metabolism, potentially through activating translocation of PKM2 into the nucleus of macrophages.

**FIGURE 5:**
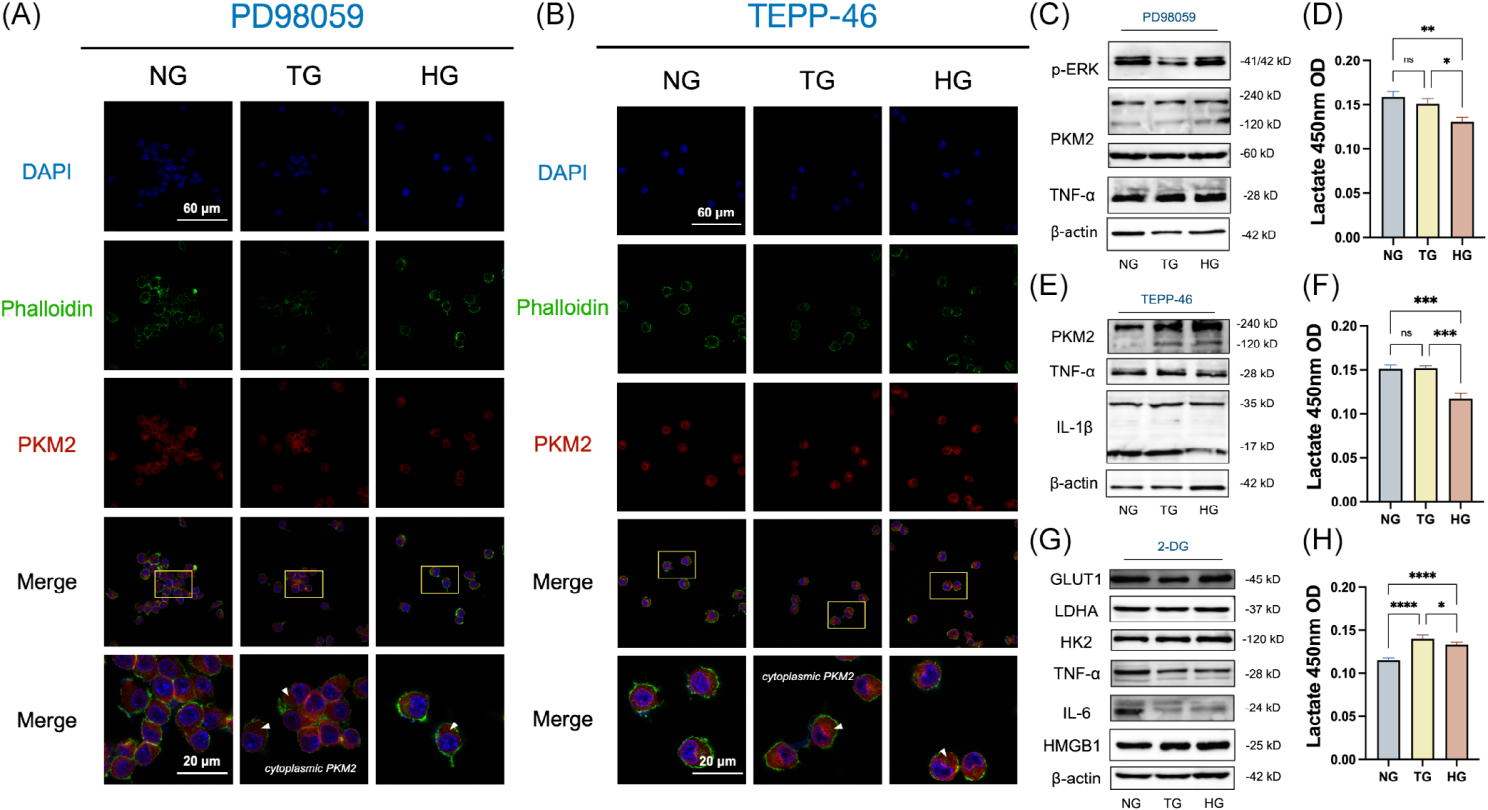
PKM2 nuclear translocation and glycolysis are mediated by ERK/PKM2. (A and B) Immunofluorescence staining for PKM2 in the iBMDM dealt with PD98059 and TEPP‐46 of NG, TG and HG. White arrows indicate the expression of PKM2 in cytoplasm. Scale bar: 20 μm. (C, E and G) Expression levels of p‐ERK, PKM2, TNF‐α, IL‐6, IL‐1β and LDHA in BMDMs dealt with PD98059, TEPP‐46 and 2‐DG separately. (D, F and H) The lactate production in macrophages were quantified. (I) Schematic diagram of the effect of ERK/PKM2 axis on macrophages dysfunction in periodontal inflammation. Values are expressed as mean ± standard deviation (*n* = 4), repeated three times. *p < 0.05; **p < 0.01; ***p < 0.001; ****p < 0.0001. NG, normoglycaemia; TG, transient hyperglycaemia; HG, hyperglycaemia.

Next, we further investigated whether the inflammation and glycolysis enhancement induced by TIH in iBMDM depended on PKM2 nuclear translocation. The phosphorylation of ERK was effectively inhibited by PD98059^22^ while TEPP‐46, a specific activator of tetrameric PKM2, was employed to suppress the activation of ERK and translocation of PKM2 in macrophages^23^ As shown in Figure 5A, C, PD98059 demonstrated significant efficacy in inhibiting nuclear translocation of PKM2 and reducing p‐ERK levels (Appendix Figure 5C–G). Furthermore, this inhibition resulted in suppression of inflammatory cytokines, glycolysis, as well as ROS production (Figure 5C, D; Appendix Figure 5A,B,H). After pre‐treatment with TEPP‐46, the nuclear accumulation of dimeric PKM2 was decreased, leading to a reduction in macrophage glycolysis levels and inflammatory cytokines levels (Figure 5B, E–F; Appendix Figure 5I–O). To further validate the involvement of glycolysis in macrophage inflammatory response, 2‐Deoxy‐D‐glucose (2‐DG) was introduced as a competitive inhibitor of glucose metabolism and suppressed glycolysis^24^ (Appendix Figure 5P,Q). Western blot assays indicated that the elevation of glucose transporter 1 (GLUT‐1), LDHA, HK2, TNF‐α, IL‐6 and HMGB1 induced by TG and HG were eliminated upon inhibition of glycolysis (Figure 5G, H; Appendix Figure 5R–W). Collectively, the above results indicate the critical role of ERK/PKM2‐mediated glycolytic rewiring in driving the inflammation response triggered by TIH.

## 4 DISCUSSION

**FIGURE 6:**
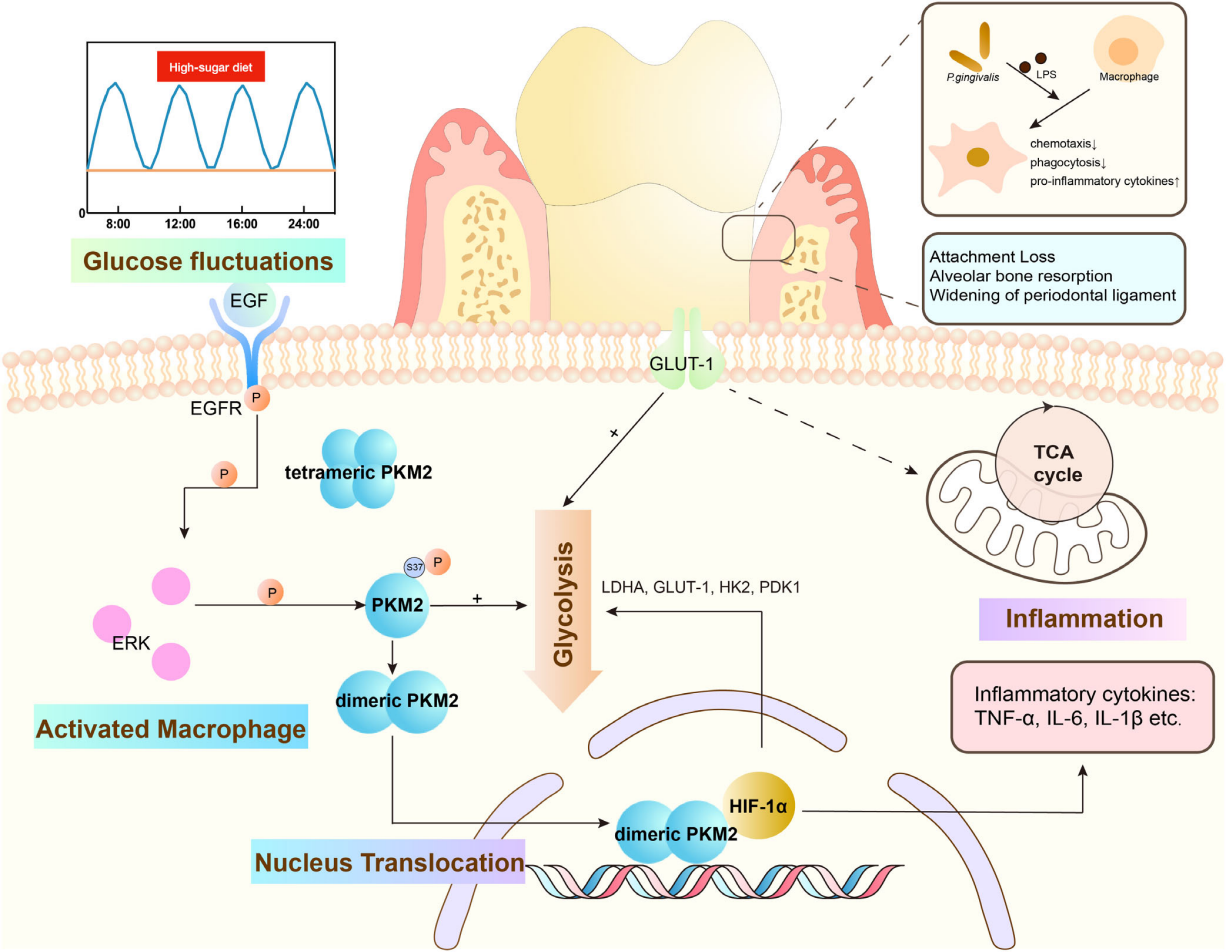
Schematic diagram of the effect of ERK/PKM2 axis on macrophages dysfunction in periodontal inflammation.

Our previous study demonstrated that hyperglycaemia triggers generalized chronic low‐level inflammation in diabetic mice, which can be alleviated by glycaemic control.^25^, ^26^ Recent advancements in the field of diabetes‐related‐inflammatory diseases have provided a deeper understanding of early glucose level intervention, as some diabetic complications may persist even after the FBG levels returned to normal.^27^, ^28^, ^29^ In current study, we utilized a TIH model on C57BL/6 mice to mimic glucose level fluctuations observed in pre‐diabetes patients. We found that TIH mice exhibited exacerbated alveolar bone resorption and attachment loss in periodontal tissues after *P. gingivalis* infection. Our findings demonstrated that TIH mediated ERK/PKM2 pathway to activate the nuclear translocation of dimeric PKM2 and induced metabolic shift towards glycolysis in macrophages, which provides insights into the development of inflammatory complications in hyperglycaemic mice (Figure 6).

Intriguingly, severe inflammation and alveolar bone resorption still existed in TIH mice despite the normalization of blood glucose levels. This phenomenon indicated that macrophages with inflammatory hyper‐reactivity may have an everlasting effect, which derived from epigenetic modifications and metabolic reprogramming.^30^, ^31^ Macrophages are essential immune cells in periodontal tissues defence, and previous researches indicated that the accumulation of dysfunctional and pro‐inflammatory macrophages was responsible for periodontal destruction in diabetic patients.^32^, ^33^ Macrophages were found to have a memory effect of metabolic environment (e.g., hypoxia^34^ hyperlipidaemia^27^ and hyperglycaemia^6^), thus induced persistent phenotype and functional change of macrophages, which attributed to epigenetic changes and metabolic reprogramming. Moreover, dysregulated macrophages can lead to metabolic‐related complications despite metabolic conditions were removed. Previous research has demonstrated that high glucose and LPS could increase the expression of M1 macrophage markers (iNOS, TNF‐α and IL‐6)^35^ Our results suggest that persistently elevated glucose levels (HG) and fluctuations in blood glucose (TG) may stimulate the secretion of TNF‐α, IL‐6 and IL‐1β in iBMDM, which emphasized the persistence of early blood glucose fluctuations in shaping macrophage inflammatory responses.

Pyruvate kinase serves as a rate‐limiting enzyme in glycolysis, facilitating the conversion of phosphoenol‐pyruvate (PEP) to pyruvate and activating the pentose phosphate pathway (PPP).^17^ The pyruvate kinase genes in mammalian cells encode for PKR, PKL, PKM1 and PKM2. While PKM1 is predominantly expressed in myocardial tissue, muscle and brain, PKM2 exhibits widespread expression across various tissues and organs. PKL and PKR are expressed in hepatocytes and erythrocytes, respectively. Structurally, PKM2 typically exists in dimeric or tetrameric form with distinct biological activities. Although dimeric PKM2 is metabolically inactive, it has been confirmed that dimeric PKM2 plays a critical role in metabolic reprogramming in inflammatory diseases.^36^ Our findings showed that sustained or transient hyperglycaemia can exacerbate inflammation and glycolysis through ERK‐dependent nuclear translocation of dimeric PKM2 in mice, with an increase in the phosphorylation level of PKM2 on serine 37 in vivo and in vitro. Except for serine, the enzymatic activity can also be regulated by multiple posttranslational modifications, including lysine acetylation and tyrosine phosphorylation. After recruiting PIN1 for cis‐trans isomerization, PKM2 binds to importin α5 and translocates into nucleus, which has been verified through co‐immunoprecipitation in previous research.^11^ The activation of ERK/PKM2 signalling may lead to macrophage dysfunction in TG, manifested by the secretion of pro‐inflammatory cytokines and impaired phagocytosis and chemotaxis abilities. The nuclear translocation of PKM2 in activated macrophages initiates hypoxia‐inducible factor 1α (HIF‐1α) (Appendix Figure 4K), thus induced an amplified level of HMGB1, TNF‐α and IL‐1β. This leads to a heightened demand for biosynthesis for proteins and energy. Augmented aerobic glycolysis efficiently meets this demand by rapidly generating sufficient energy. Additionally, nuclear PKM2 serves as a transcriptional co‐activator to upregulate the expression of glycolysis‐related enzymes, consequently boosting glycolytic activity. As the end product of glycolysis, lactate accumulated in activated macrophages. PKM2‐mediated aerobic glycolysis promoted the release of HMGB1, highlighting the significance of targeting aerobic glycolysis in therapeutic interventions for inflammatory diseases^37^

PD98059, an inhibitor of ERK phosphorylation, substantially inhibited the formation and nuclear translocation of dimer PKM2 and reversed the production of inflammatory cytokines, as well as enhancing glycolysis in vitro. TEPP‐46 functions as a selective activator of PKM2 (with minimal effect on PKM1, PKL and PKR), which facilitates the development of tetramers through inhibiting nuclear translocation of PKM2^38^ After incubation with TEPP‐46, the glycolysis and release of pro‐inflammatory cytokines was attenuated. However, it is worth noting that the ERK inhibitor blocked dimeric PKM2 more effectively than TEPP‐46, suggesting ERK/PKM2 mediated PKM2 nuclear translocation. Furthermore, treatment with 2‐DG effectively eliminated TIH‐induced inflammation in macrophages, indicating that TIH induced ERK‐phosphorylation‐dependent PKM2 nuclear translocation to regulate glycolytic reprogramming.^39^

In summary, the relationship between transient hyperglycaemia and inflammation diseases is intricate. Our results suggested that TIH activated ERK/PKM2 pathway to facilitate glycolytic rewiring and amplify inflammation in periodontitis. There are still a few limitations of our study that gene knock‐out mice, which are beneficial for illustrating detailed mechanisms, are not available in this study. Furthermore, several other glucose metabolism pathways have not been investigated. Within the limitation of the study, our findings provided a clinically relevant rationale for understanding ERK/PKM2‐mediated macrophage inflammation in glucose variation‐related inflammation lesions in periodontal tissues, which alerted the significance of early control of blood glucose.

## AUTHOR CONTRIBUTIONS

Yuezhang Sun contributed to conception, performed animal and cellular experiments, drafted and critically revised the manuscript; Aimin Cui conducted molecular experiments, drafted and critically revised the manuscript; Hao Dong performed animal experiments; Lulingxiao Nie and Ziqi Yue provided technical and theoretical help; Jiao Chen contributed to data collection and analysis; Jian Wang and Wai keung Leung drafted and critically revised the manuscript; Qi Wang conceived this study, analysed and interpreted the data, raised the funding, drafted and critically revised the manuscript. All authors gave their final approval and agree to be accountable for all aspects of the work.

## FUNDING INFORMATION

This work was supported by the National Natural Science Foundation of China (81870779 to Qi Wang), Natural Science Foundation of Sichuan Province (2024NSFSC0548), and the Chengdu Science and Technology Program (2022‐YF05‐01760‐SN to Qi Wang). The funding bodies played no role in the design of the study, the collection, analysis and interpretation of the data, and the writing of the manuscript.

## CONFLICT OF INTEREST STATEMENT

The authors declare no conflicts of interest.

## Supporting information


**Data S1.** Supporting Information.

## Data Availability

The data that support the findings of this study are available from the corresponding author upon reasonable request.
