# Supplementary material for: Intermittent hyperglycaemia induces macrophage dysfunction by extracellular regulated protein kinase‐dependent PKM2 translocation in periodontitis
Source: Cell Prolif. 2024 May 24;57(10):e13651. doi: 10.1111/cpr.13651 (PMC11471441; doi:10.1111/cpr.13651)
Supplement: Supplementary file 1 — Data S1. Supporting Information. [file CPR-57-e13651-s001.docx]

**Intermittent hyperglycemia-induced ERK-dependent PKM2 translocation in macrophages fuels periodontitis**

Yuezhang Sun^1,†^, Aimin Cui^1,†^, Hao Dong^1^, Lulingxiao Nie^1^, Ziqi Yue^1^, Jiao Chen^1^, Wai Keung Leung^2^, Jian Wang^1^, Qi Wang^1,*^

**Materials and Methods**

1. Bacterial infection

*Porphyromonas gingivalis* (*P.gingivalis*, ATCC33277) was obtained from the State Key Laboratory of Oral Diseases of Sichuan University, which is considered as the most common pathogen suspected of inducing periodontitis. *P.gingivalis* was grown in sheep-blood agar with hemin/vitamin K1 anaerobically for a week. After three passages of cultivation, the pure culture was inoculated in liquid brain heart infusion broth medium and cultured for 48h at 37°C. The C, TIH, and D group mice was infected orally with *P.gingivalis* (a total of 10^9^ live bacterial were dispersed in 100 μL phosphate-buffered saline containing 2% carboxymethylcellulose) for 4 weeks at 2 d intervals.

2. Micro-CT analysis

The mandibular bones of all groups were scanned by a μCT50 micro-CT system (SCANCO Medical, Bruettisellen, Switzerland) at 8μm intervals. Three-dimensional (3D) reconstruction was captured by SCANO Visualizer to analyze the periodontal bone loss. Given that the three molar teeth of the mandible are not in the same straight line, the images are selected with maximum projected area of crown and root structures of three molars for standardization. The alveolar bone was determined as the region of interest (ROI) to calculate bone loss area and the length of cemento-enamal junction to alveolar bone crest (CEJ-ABC). The measurements are expressed in mm and mm^2^. As previously described, dual-channel analysis method was applied based on the different densities of the 3D reconstructed mandibular images. Specifically, one channel was selected for the crown (PET 20) and another for the remaining parts (Heat). The area of density reduction below the crown indicated bone loss. Furthermore, X-ray observation of bone resorption in the furcation area demonstrated variations in alveolar bone loss severity among different groups. According to the ROI, bone volume/total volume (BV/TV) and trabecular separation (Tb.Sp) were calculated for quantification.

3. Histological and immunohistochemical analysis

The maxillary samples were decalcified in 10% EDTA solution (G1105, Servicebio), with the solution changed every 3 days for 30 d. Samples were embedded in paraffin and incised (4 μm) along the long axis of the tooth with a disposable microtome. Tissue sections containing three complete molars were selected for staining using an optical microscope. Sections were incubated with primary antibodies against IL-1β (HA601036, Huabio), IL-6 (R1412-2, Huabio) and TNF-α (ER65189, Huabio). The sections were incubated with secondary antibody at room temperature for 30 min after washing with PBS. The samples were imaged under Slideview VS200 (Olympus). The images were analyzed by the ImageJ software. The integrated option density (IOD) value and area of each image were measured, followed by the calculation of the mean density (mean density = IOD/area), which represents the concentration of the target protein per unit area. Finally, the mean density of 5 random areas of each sample was taken as the value of this sample.

4. Immunocytochemistry

Cells were fixed with 4% PFA for 15 min and incubated with 0.1% Triton X-100 for 10 min at room temperature, then were blocked in PBS with 4% (v/v) goat serum and 1% (v/v) glycerol for 1 h. Cells were incubated with primary antibody against PKM2 (R1603-5, Huabio), ERK (ET1601-29, Huabio), and p-ERK (ET1610-13, Huabio) overnight at 4°C, followed by incubation with anti-rabbit secondary antibody for 1 h and anti-Phalloidin for 10 min at 37°C. After nuclear staining with DAPI, images were captured by laser confocal microscopy FV3000 (Olympus).

5. Lactate and LDH activity measurement

The serum was obtained from the blood samples of mice by centrifugation at 3000 rpm for 5 min at 4°C. The mice serum and cells were separated as described to evaluated lactate levels by Lactate Fluorometric Assay Kit (KTB1100, Abbkine) and Lactate Dehydrogenase (LDH) Assay Kit (KTB1110, Abbkine) according to the manufacturer’s protocols with replicates. The absorbency of all samples was detected at 450 nm by a microplate reader (Bio-rad, USA).

6.Western blot

The soft tissues around the mandibular molars were gently extracted to obtain the protein from mouse gingival tissues. Total proteins were extracted from cells and mice gingival tissues according to the manufacturer’s protocol. The cell lysate was then centrifuged at 13,000 rpm at 4°C for 15 min. The supernatant was mixed with 5× sample loading buffer (P1015, Solarbio) in a ratio of 4:1. Sample were subjected to electrophoresis on 4-15% sodium dodecyl sulfate-polyacrylamide (SDS–PAGE). The proteins were transferred to nitrocellulose membranes by electro-blotting, which were blocked in 5% milk (BD) and incubated overnight at 4°C with primary antibodies against β-actin, PKM2 (R1603-5, Huabio), p-PKM2 s37 (AF7231, Affinity), ERK (ET1601-29, Huabio), p-ERK (ET1610-13, Huabio), TNF-α (11948, Cell signaling technology), IL-6 (12912, Cell signaling technology), HMGB1 (ET1601-2, Huabio), GLUT-1 (73015, Cell signaling technology), HK2 (2867, Cell signaling technology), PDK1 (ET1704-66, Huabio), importin α5 (18137-1-AP, proteintech), and PIN1 (R25374, Zenbio) and followed with secondary antibodies for 1 hour at room temperature. Membranes were incubated with electrochemiluminescence (ECL) substrate (P0018M, Beyotime). The signals were visualized using a Bio-Rad system (Bio-Rad Laboratories, Shanghai, China). For quantification, phosphorylated proteins were standardized based on the levels of total protein (phosphorylated + non-phosphorylated), and other proteins were achieved by normalizing to β-actin.

7. ROS measurement

Reactive Oxygen Species (ROS) was measured using a ROS Fluorometric Assay Kit (E-BC-K138-F, Elabscience) according to the manufacturer’s instructions. iBMDM were seeded at a density of 105 cells per well in a 6-well plate. Cells were cultured with normoglycemia (NG), transient intermittent hyperglycemia (TG), and hyperglycemia (HG) respectively, followed by LPS stimulating. Subsequently, cells were incubated with 2,7-dichlorofuorescin diacetate (DCFH-DA) probe for 30 min in the dark at 37°C. After washing three times with serum-free DMEM, we detected the ROS intensity at 530 nm with a fluorescent microplate reader. The experiment was repeated three times.

8. Transwell migration assay

Transwell migration assay for cells were conducted according to the previous protocol ^[1]^. 1 × 10^5^ cells/well were suspended in FBS-free high-glucose DMED and seeded into the upper chamber of an 8 μm pore insert (3428, Corning) in a 6-well plate. The lower chamber was supplemented with 1.5mL DMEM. Following incubation at 37°C in a humidified incubator with a CO2 concentration of 5% for 24 hours, the chambers were fixed using a solution of 4% PFA and stained with crystal violet (0.1%) at room temperature for a duration of 30 minutes. Cells on the upper surface of filter membranes were removed with a wet cotton swab. After washing with PBS for three times, images were captured with an optical microscope to observe the migration of iBMDM, and subsequently compared to those of the control group.

9. Latex beads phagocytosis

To explore the phagocytosis of iBMDM, red fluorescence latex beads (L2778, Sigma-Aldrich) were initially mixed with 1% BSA at a ratio of 1:100 and subsequently incubated at 37°C for 30 minutes in the dark. Five minutes of ultrasonic treatment was employed before use. A total volume of 2 ml latex beads was added into the culture medium and incubated at 37°C for 1.5 hours. Cells were scraped and washed twice by centrifugation at 1500 rpm/5 min, followed by resuspension in 500 μl of pre-chilled PBS. The cell suspension was subsequently assessed using the Attune Nxt flow cytometer (Thermo Fisher Scientific). Results were analyzed by FlowJo software (version 10.6.2) ^[2]^. Moreover, the images of macrophages swallowing red fluorescence latex beads were captured with a laser confocal microscopy FV3000 (Olympus).

**Appendix Figures**:


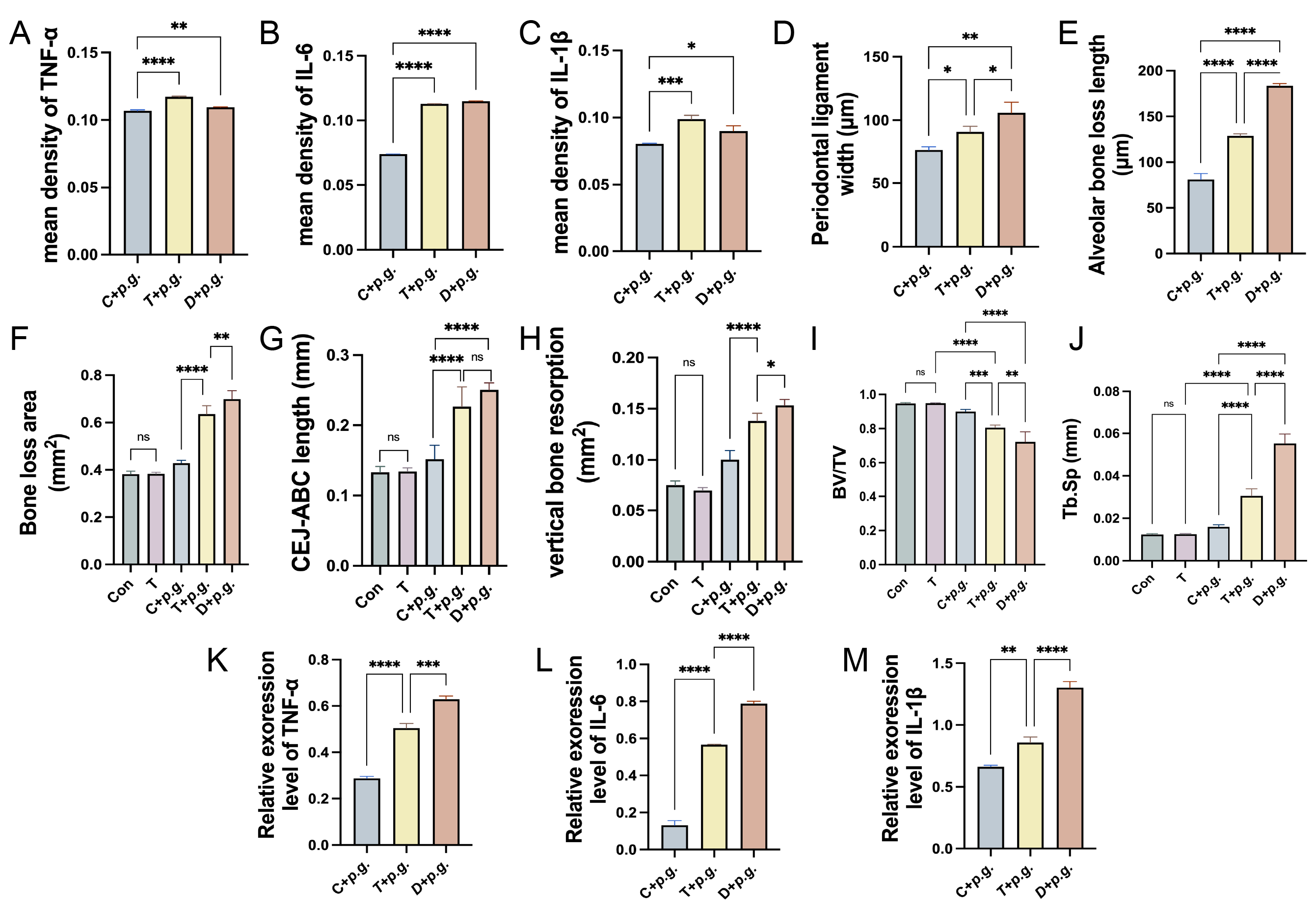


**Appendix figure 1**. A, B, and C) IHC staining show the expression levels of TNF-α, IL-6, and IL-1β respectively in periodontal tissues. D, E) H&E staining demonstrate the alveolar bone loss and periodontal ligament width in each group. F, G, and H) The analysis of Micro-CT shows the level of bone resorption and attachment loss.I, J) Quantification of bone volume/total volume (BV/TV) and Tb.Sp (trabecular separation). K-M) The relative expression level of TNF-α, IL-6, and IL-1β protein is shown in bar histograms. Data are expressed as mean ± standard deviation (*n*=5). ns, no significance. **p*<.05. ***p*<.01. *****p*<.0001. Con, normoglycemic mice; T, transient intermittent hyperglycemic mice; C+*P.g.*, *P.gingivalis* infection-normoglycemic mice; T+*P.g.*, *P.gingivalis* infection- transient intermittent hyperglycemic mice; D+*P.g.*, *P.gingivalis* infection-diabetic mice.


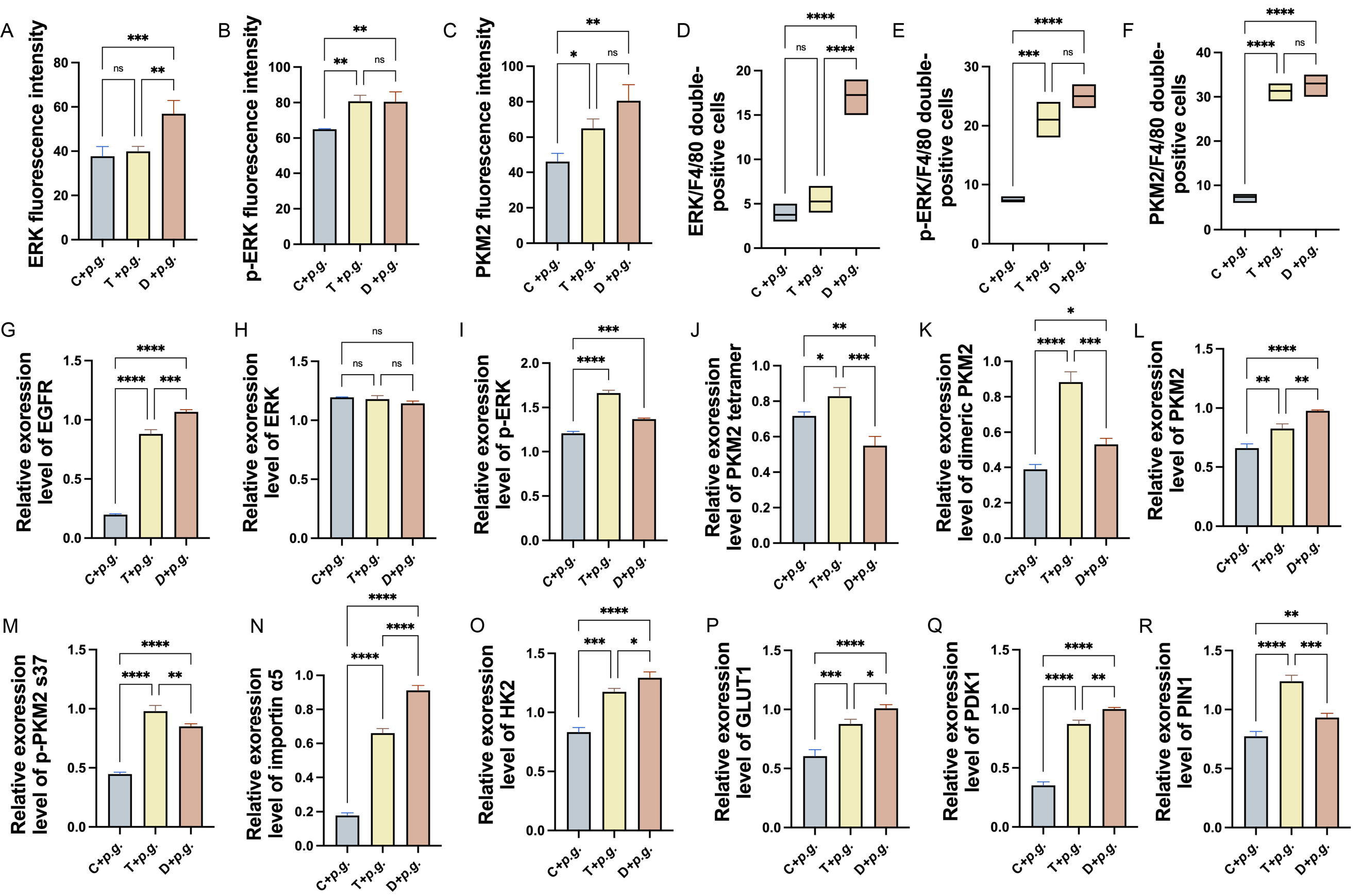


**Appendix figure 2**. A, B, and C) Immunofluorescence staining reveal the mean fluorescence intensity of ERK, p-ERK, and PKM2 respectively in periodontal tissues. D, E, and F) Immunofluorescent staining validation of the co-staining of ERK/p-ERK/PKM2 and F4/80-positive cells. G-R) The relative expression level of EGFR, ERK, p-ERK, PKM2, p-PKM2, importin α5, HK2, GLUT1, PDK1, and PIN1 protein is shown in bar histograms. Data are expressed as mean ± standard deviation (*n*=4). ns, no significance. **p*<.05. ***p*<.01. *****p*<.0001. C+*P.g.*, *P.gingivalis* infection-normoglycemic mice; T+*P.g.*, *P.gingivalis* infection- transient intermittent hyperglycemic mice; D+*P.g.*, *P.gingivalis* infection-diabetic mice.


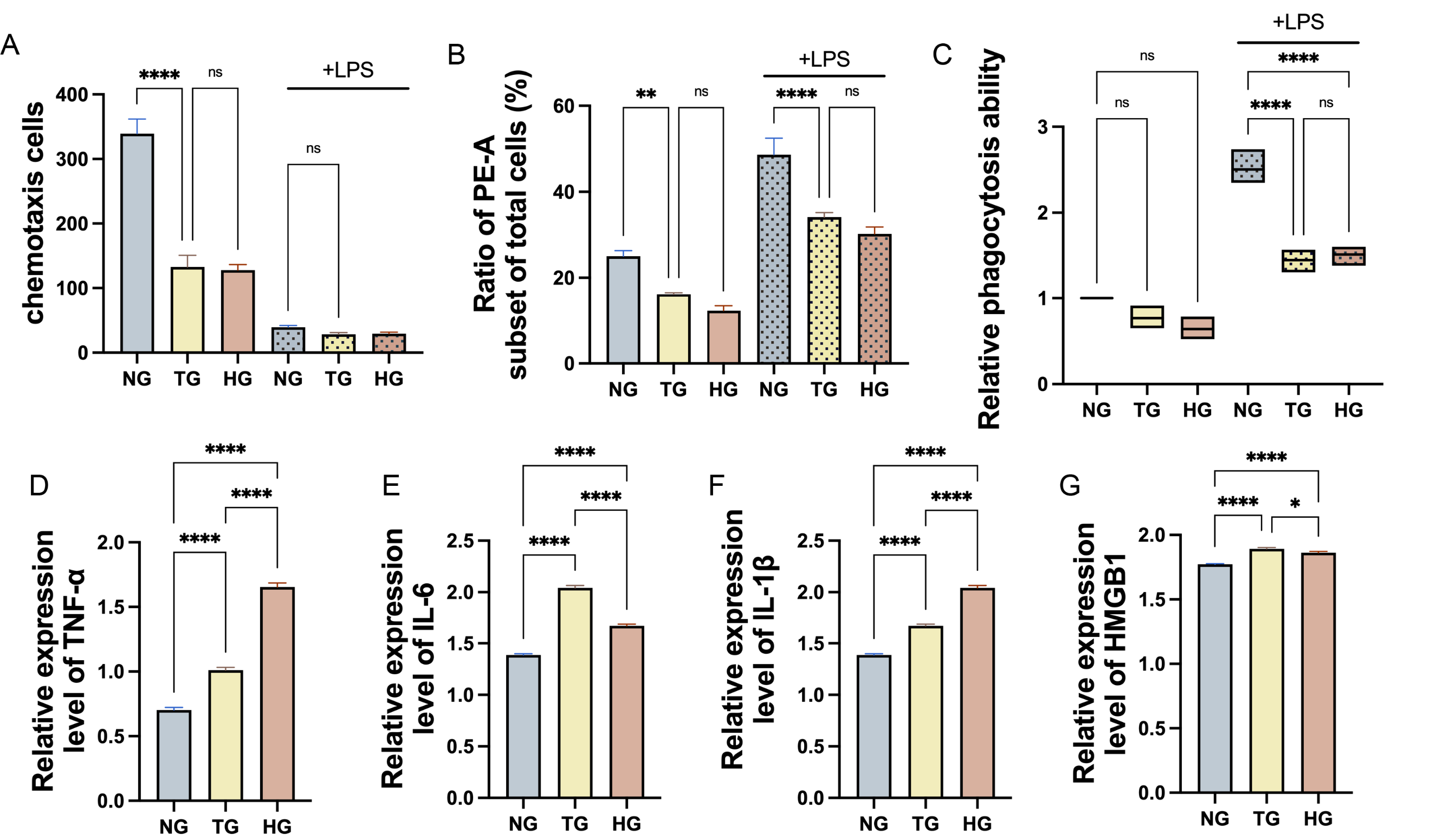


**Appendix figure 3**. A) The evaluation of chemotaxis capability of different groups. B, C) Flow cytometry and immunofluorescence staining showed the phagocytosis ability of different groups. D-G) Expression level of inflammatory cytokines (TNF-α, IL-6, IL-1β, and HMGB1) assayed by western blot for iBMDM. Data are expressed as mean ± standard deviation, repeated three times. ns, no significance. **p*<.05. ***p*<.01. ****p*<.001. *****p*<.0001. NG, normoglycemia; TG, transient hyperglycemia; HG, hyperglycemia.


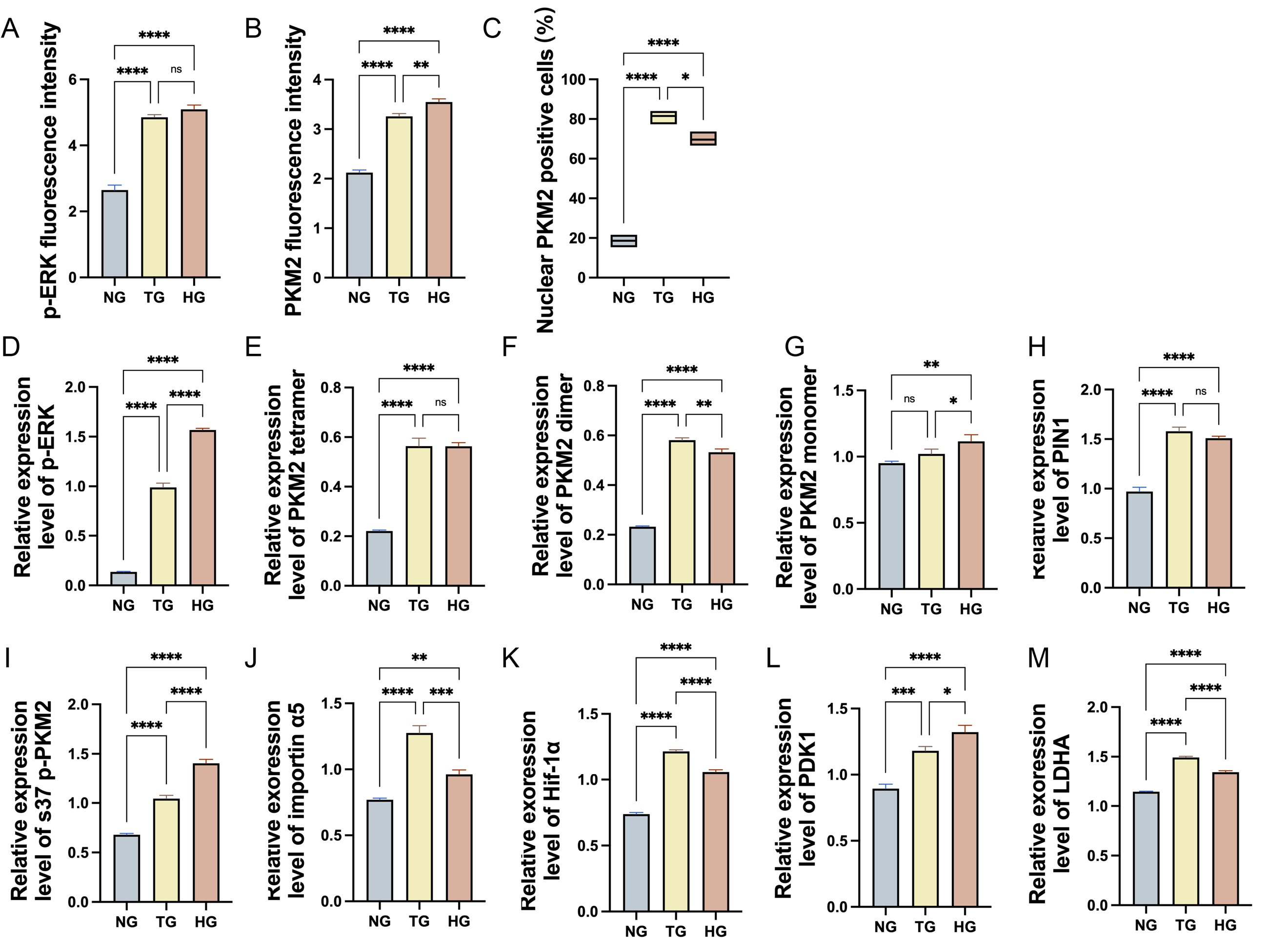


**Appendix figure 4**. A, B) Immunofluorescence staining demonstrated the expression level of p-ERK and PKM2 in macrophages. C) Nuclear-PKM2 positive cells ratio detected by immunofluorescence staining. D-M) The relative expression level of p-ERK, PKM2, p-PKM2, importin α5, Hif-1α, PDK1, and LDHA protein is shown in bar histograms. Data are expressed as mean ± standard deviation, repeated three times. ns, no significance. **p*<.05. ***p*<.01. ****p*<.001. *****p*<.0001. NG, normoglycemia; TG, transient hyperglycemia; HG, hyperglycemia.


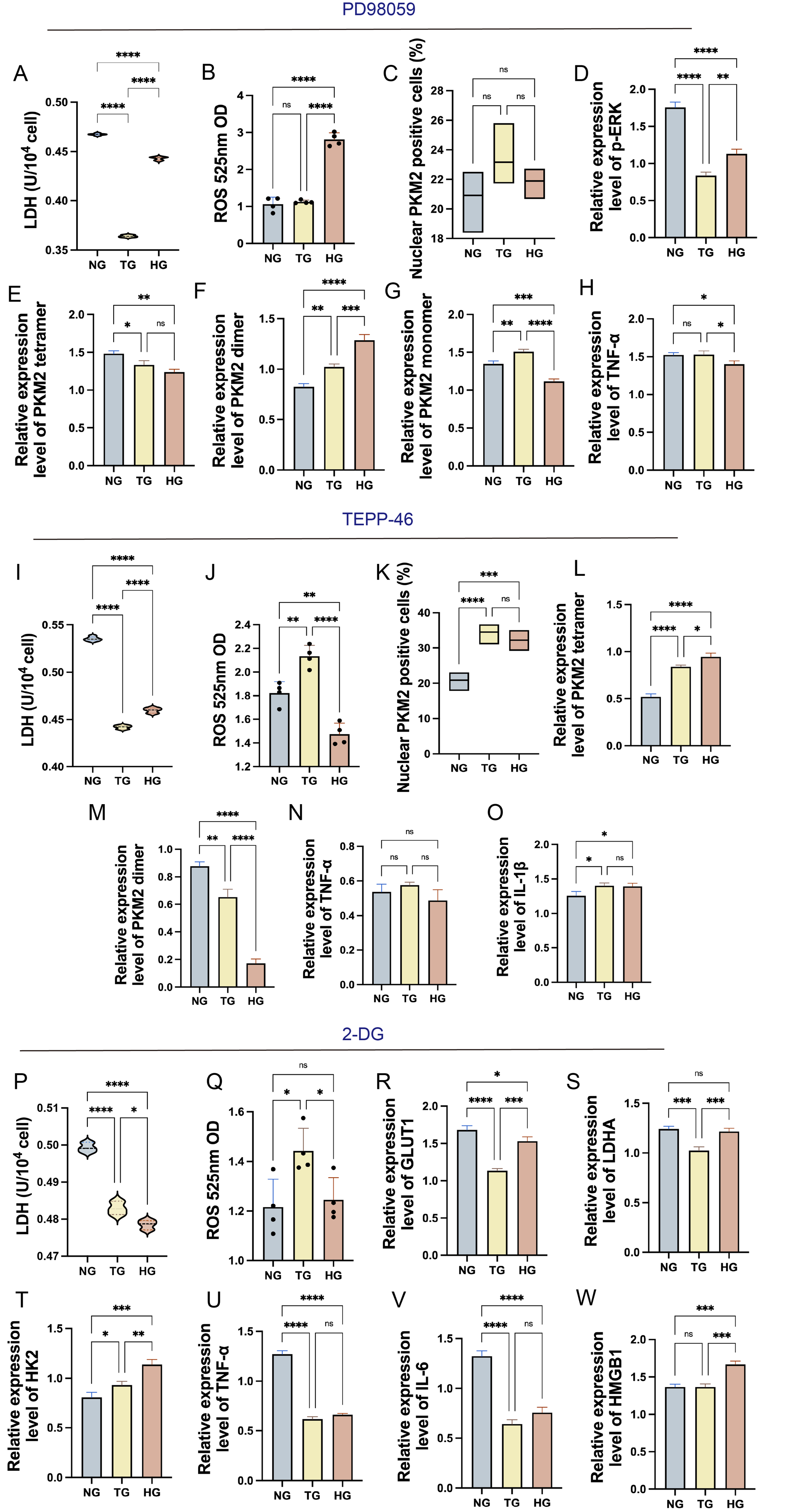


**Appendix figure 5**. A, I, and P) The LDH enzyme activity evaluated by an assay kit for iBMDM cultured with PD98059, TEPP-46, and 2-DG. B, J, and Q) The reactive oxygen species production detected with an assay kit for cells cultured with PD98059, TEPP-46, and 2-DG. C, K) Nuclear-PKM2 positive cells ratio evaluated by immunofluorescence staining. D-H, L-O, and R-W) The relative expression level of p-ERK, PKM2, TNF-α, IL-1β, GLUT1, LDHA, HK2, IL-6, and HMGB1 protein is shown in bar histograms. Data are expressed as mean ± standard deviation, repeated three times. ns, no significance. **p*<.05. ***p*<.01. ****p*<.001. *****p*<.0001. NG, normoglycemia; TG, transient hyperglycemia; HG, hyperglycemia.


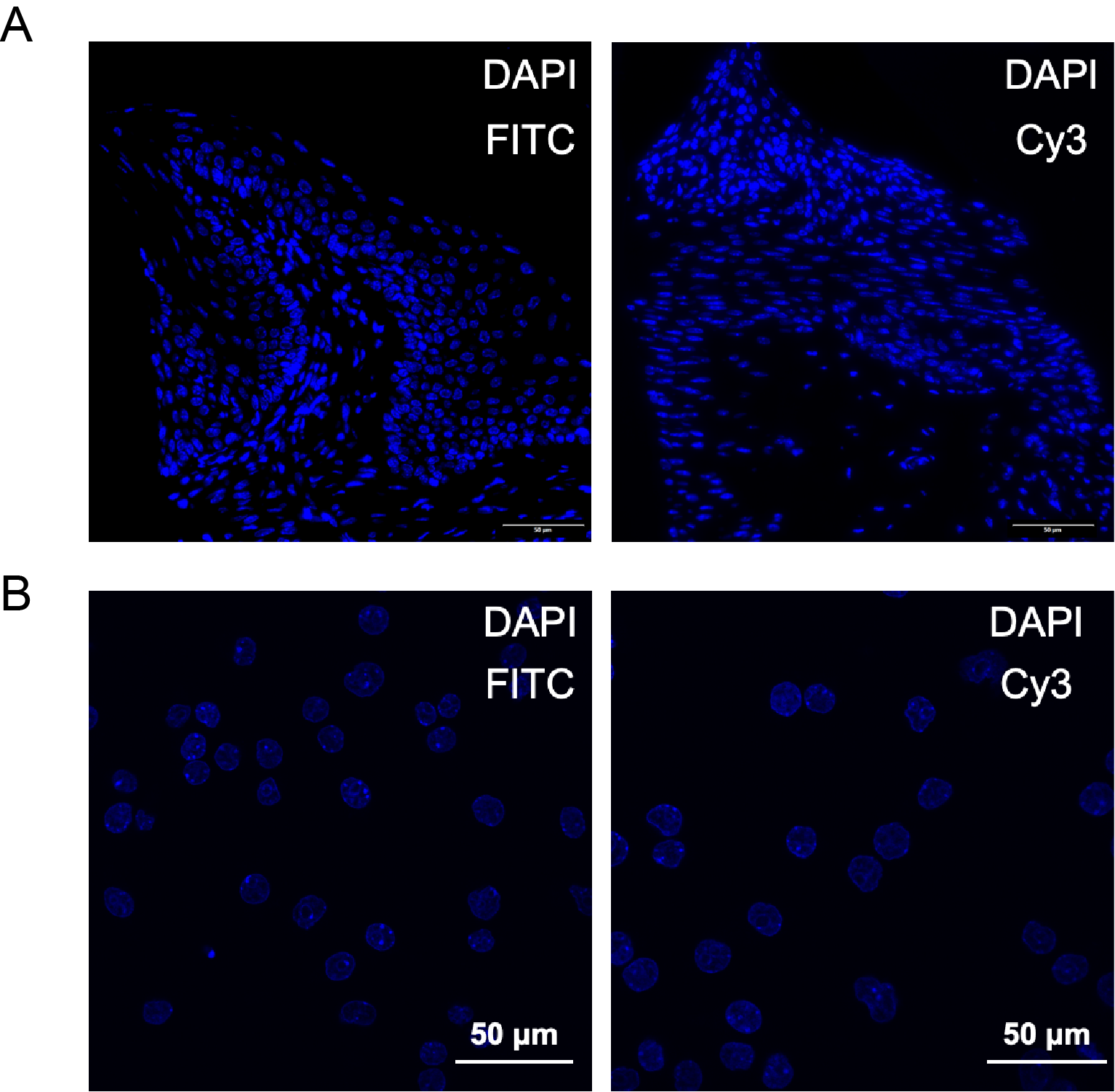


**Appendix figure 6**. Immunofluorescence staining of mice gingiva tissues (A) and iBMDM (B) was performed with secondary antibody controls without primary antibody. *Scale bar: 50μm*.

References

1. Nie L, Zhao P, Yue Z, Zhang P, Ji N, Chen Q, Wang Q. 2021. Diabetes induces macrophage dysfunction through cytoplasmic dsdna/aim2 associated pyroptosis. J Leukoc Biol. 110(3):497-510.

2. Yue Z, Nie L, Ji N, Sun Y, Zhu K, Zou H, Song X, Chen J, Wang Q. 2023. Hyperglycaemia aggravates periodontal inflamm-aging by promoting setdb1-mediated line-1 de-repression in macrophages. J Clin Periodontol. 50(12):1685-1696.
